# Supplementary material for: Prognostic Value of Holter Monitoring in Light Chain Amyloidosis
Source: J Clin Med. 2023 Dec 1;12(23):7457. doi: 10.3390/jcm12237457 (PMC10707322; doi:10.3390/jcm12237457)
Supplement: Supplementary file 1 [file jcm-12-07457-s001.zip › jcm-2652144-supplementary.pdf]

SUPPLEMENTARY MATERIAL

Sun et al. Prognostic value of Holter monitoring in light chain amyloidosis

Contents:

I: Supplementary methods

Sample size calculation **(P2)**

II: Supplementary references (P3)

III: Supplementary tables

Table S1: Overall Survival in CA/non-CA Patients**(P4)**

Table S2: Overall survival of CA Patients based on treatment strategies (n=110) **(P4)**

IV: Supplementary figures

Figure S1: Kaplan-Meier survival estimates for OS based on the presence or absence of CA **(P5)**

Figure S2: Kaplan-Meier survival estimates for OS in CA patients based on their treatment strategies **(P5)**

I: Supplementary methods

Sample size calculation

The sample size calculation for the clinical prediction model was based on the estimated risk of the primary endpoint events in the target population, the number of predictor variables to be included, and the expected goodness-of-fit of the model measured by Cox-Snell R^2[1].

The formula used for calculation was:

$$n = P/(S-1)ln\left(1-\frac{R^2cs}{S}\right)$$

Based on previous literature reports [2,3], the incidence rate of overall mortality in the AL amyloidosis population was approximately 20%. The median follow-up time in this study was 2 years, with Cox-Snell R^2 set at 0.20. Considering the inclusion of 3 variables, the calculated required sample size was 120 patients.

The sample size calculation for this study was performed using R software version 4.1.2 (R Foundation for Statistical Computing, Vienna, Austria) pmsampsize function [4].

II: Supplementary References:

1. Riley, R. D.; Ensor, J.; Snell, K. I. E.; Harrell, F. E.; Jr, Martin, G. P.; Reitsma, J. B.; Moons, K. G. M.; Collins, G.; van Smeden, M. Calculating the Sample Size Required for Developing a Clinical Prediction Model. BMJ 2020, 368, m441. https://doi.org/10.1136/bmj.m441.

2. Kastritis, E.; Palladini, G.; Minnema, M. C.; Wechalekar, A. D.; Jaccard, A.; Lee, H. C.; Sanchorawala, V.; Gibbs, S.; Mollee, P.; Venner, C. P., et al. Daratumumab-Based Treatment for Immunoglobulin Light-Chain Amyloidosis. N. Engl. J. Med. 2021, 385 (1), 46–58. https://doi.org/10.1056/NEJMoa2028631.

3. Sidana, S.; Tandon, N.; Brady, P. A.; Grogan, M.; Gertz, M. A.; Dispenzieri, A.; Lin, G.; Dingli, D.; Buadi, F. K.; Lacy, M. Q.; et al. Prognostic Significance of Holter Monitor Findings in Patients with Light Chain Amyloidosis. Mayo Clin. Proc. 2019, 94 (3), 455–464. https://doi.org/10.1016/j.mayocp.2018.08.039.

4. Ensor, J.; Martin, E. C.; Riley, R. D. Pmsampsize: Calculates the Minimum Sample Size Required for Developing a Multivariable Prediction Model, 2022. https://cran.r-project.org/web/packages/pmsampsize/index.html (accessed 2023-10-19).

III: Supplementary Tables:

Table S1: Overall Survival in CA/non-CA Patients

**Variable**	**0-5 years OS (95% CI）**	**p value**	**Median OS (95% CI)**	**p value**
CA patients (n=110)	55.5(49.7-61.3)	**0.070**	68.7	**0.045**
non-CA patients (n=27)	73.5(63.1-83.9)		NR*	

Notes: CA: cardiac amyloidosis; OS: overall survival; NR: not reached.
*: The overall mortality rate did not reach 50% at the end of the follow-up.

Table S2: Overall survival of CA Patients based on treatment strategies (n=110)

**Variable**	**0-5 years OS (95% CI）**	**p value**	**Median OS (95% CI)**	**p value**
Daratumumab-based therapy (n=28)	85.7(79.1-92.3)	0.215	85.7(79.1-92.3)	0.215
Other therapies (n=82)	52.6(46.5-58.7)		42.7(35.5-49.9)	

Note: CA: cardiac amyloidosis; OS: overall survival.

IV: Supplementary Figures:

Figure S1: Kaplan-Meier survival estimates for OS based on the presence or absence of CA

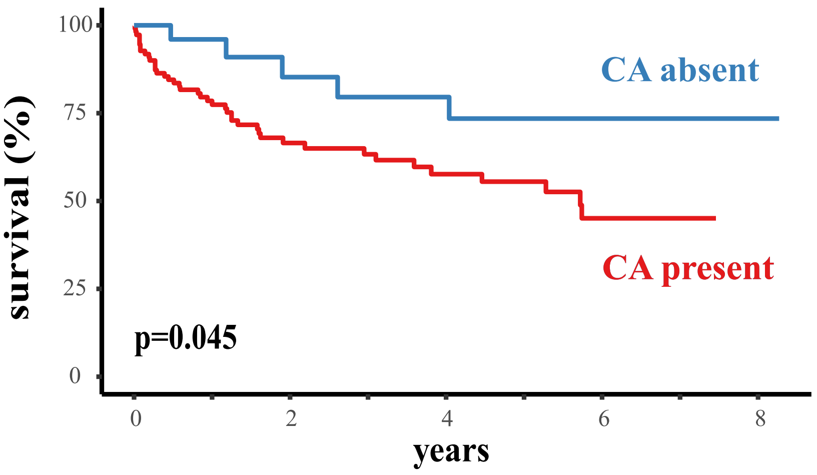

Note: CA: cardiac amyloidosis; OS: overall survival.

Figure S2: Kaplan-Meier survival estimates for OS in CA patients based on their treatment strategies

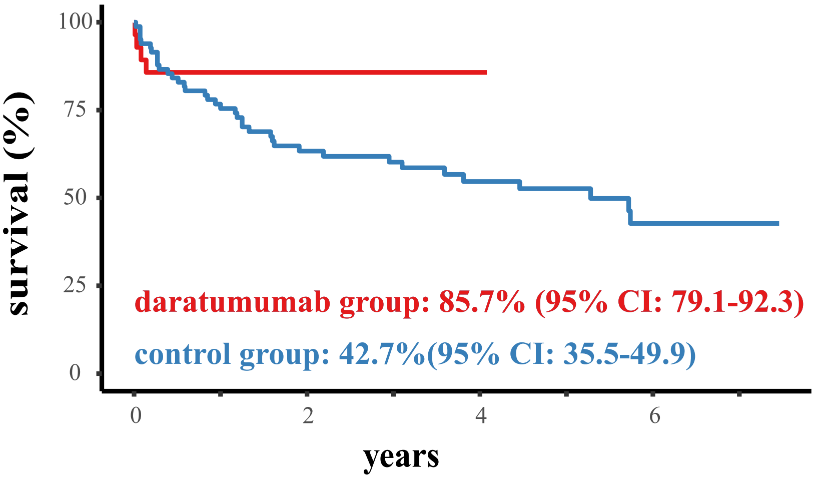

Note: CA: cardiac amyloidosis; OS: overall survival.
